# First-Principles Study of a MoS_2_-PbS van der Waals Heterostructure Inspired by Naturally Occurring Merelaniite

**DOI:** 10.3390/ma14071649

**Published:** 2021-03-27

**Authors:** Gemechis D. Degaga, Sumandeep Kaur, Ravindra Pandey, John A. Jaszczak

**Affiliations:** Department of Physics, Michigan Technological University, Houghton, MI 49931, USA; gdegaga@mtu.edu (G.D.D.); sumandek@mtu.edu (S.K.); jaszczak@mtu.edu (J.A.J.)

**Keywords:** merelaniite, vdW heterostructure, MoS_2_, PbS, carrier mobility

## Abstract

Vertically stacked, layered van der Waals (vdW) heterostructures offer the possibility to design materials, within a range of chemistries and structures, to possess tailored properties. Inspired by the naturally occurring mineral merelaniite, this paper studies a vdW heterostructure composed of a MoS_2_ monolayer and a PbS bilayer, using density functional theory. A commensurate 2D heterostructure film and the corresponding 3D periodic bulk structure are compared. The results find such a heterostructure to be stable and possess p-type semiconducting characteristics. Due to the heterostructure’s weak interlayer bonding, its carrier mobility is essentially governed by the constituent layers; the hole mobility is governed by the PbS bilayer, whereas the electron mobility is governed by the MoS_2_ monolayer. Furthermore, we estimate the hole mobility to be relatively high (~10^6^ cm^2^V^−1^s^−1^), which can be useful for ultra-fast devices at the nanoscale.

## 1. Introduction

Two-dimensional (2D) materials are celebrated among the scientific community, due to the unparalleled exquisite properties compared to their bulk counterparts, arising from the quantum confinement effects. Some of the examples of 2D materials include graphene, h-BN, transition metal dichalcogenides (TMDs), phosphorene, MXenes, etc., which have been explored for novel device applications [1,2,3,4,5,6,7]. The possibility of synthesis of vertically stacked 2D structures, in which the consecutive layers interact through weak van der Waals forces called van der Waals (vdW) heterostructures, can give rise to nanomaterials with desired properties [8,9,10]. The vdW heterostructures present unique electronic properties due to the incommensurate and modulating nature of their structural configurations along the crystallographic *a*- and *b*-directions. Despite their fascinating and attractive properties, only a few groups have so far succeeded in the synthesis of such heterostructures [11,12,13,14,15,16,17].

Naturally occurring vdW structures such as cylindrite and franckeite belong to a family of complex, misfit, layered sulfide minerals. These materials are built from the vertically stacked alternating layers of pseudo-quadratic (referred to as *Q*) and pseudo-hexagonal (referred to as *H*) layers [18,19,20,21]. In general, the *Q* and *H* layers are a (100) slab of the rock-salt-type structure and a *CdI*_2_-type layer, respectively, and the interlayer interaction is primarily dominated by the vdW interactions.

Theoretical and experimental investigations of the 2D heterostructures derived from these incommensurate layered sulfides are still at the stage of infancy. A recent theoretical study of MoS_2_-CdS heterostructure reported its enhanced photocatalytic activity [22]. Heterostructures of alternating SnS_2_-based (*H*) and PbS-based (*Q*) layers have been synthesized by exfoliation of bulk natural franckeite [15,16]. Spectroscopic measurements found the ultrathin franckeite nanosheet to be a p-type material with an estimated bandgap of ≈0.7 eV. Note that the PbS-based *Q* layers in the franckeite structure are with non-isometric unit-cells and thus structurally different from the ones directly derived from the PbS cubic bulk structure [23]. The *Q* layer is composed of four atomic layers of sulfide compounds with the formula MX, where M = Pb^2+^, Sn^2+^ or Sb^3+^ and X = S. The *H* layer consists of octahedrons of disulfide compounds with the formula MX_2_, where M = Sn^4+^ or Fe^2+^ and X = S [16]. 

**Figure 1:**
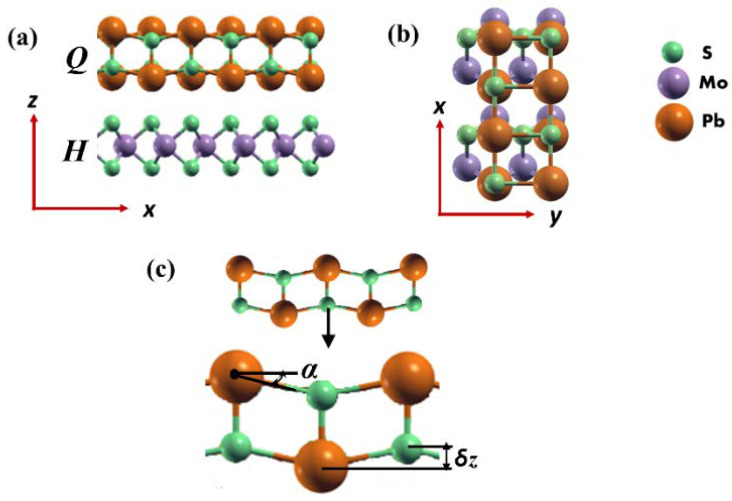
(**a**) Side view (c-axis vertical), (**b**) top view of the MoS_2_-PbS heterostructure, composed of *H* (i.e., MoS_2_ monolayer), and *Q* (i.e., PbS bilayer) layers. (**c**) The buckling parameters of the *Q* (PbS-like bilayer) layer. Color code: Mo (grey), Pb (orange), and S (green).

Following the success of fabricating 2D heterostructures from bulk franckeite, we here focus on 2D and bulk MoS_2_-PbS heterostructures that are inspired by, but simplified from another naturally occurring member of the cylindrite group, the relatively newly discovered mineral merelaniite (Mo_4_Pb_4_VSbS_15_). Merelaniite is composed predominantly of a stacking of a MoS_2_-based monolayer (*H*) incommensurately aligned with a PbS-based bilayer (*Q*) [24]. Both the *H* and *Q* layers of merelaniite are triclinic with space group *C*1. The unit cell parameters for the *H* layer are: *a* = 5.547(9) Å; *b* = 3.156(4) Å; *c* = 11.91(1) Å; α = 89.52(9); β = 92.13(5); γ = 90.18(4). For the *Q* layer, the parameters are: *a* = 5.929(8) Å; *b* = 5.961(5) Å; *c* = 12.03(1) Å; α = 91.33(9); β = 90.88(5); γ = 91.79(4). Note that the *a* and *b* directions are parallel to the layers, and *c* is the stacking direction of the *H* and *Q* layers. In this study, the structure and chemistry are simplified to be coherently aligned *H* and *Q* layers, composed solely of MoS_2_ and PbS, respectively, with a minimally sized commensurate periodic supercell in the *a-b* plane, as displayed in Figure 1. Specifically, we investigated the stability and electronic properties of this 2D vdW heterostructure employing density functional theory. The formation of such layered PbS precipitates along the (001) plane of molybdenite (MoS_2_) mineral was recently reported, thereby suggesting the existence of MoS_2_-PbS heterostructure [25]. We also calculate its carrier mobility to assess its applicability in next-generation devices based on 2D materials. We note that 2D vdW heterostructures based on the natural mineral franckeite have been proposed as candidate materials for optoelectronic devices [17,26].

## 2. Computational Model

Electronic structure calculations based on density functional theory (DFT) were performed employing the Perdew-Burke-Ernzerhof (PBE) functional for exchange and correlation [27]. To account for the vdW long-range interaction between the constituent layers, a posteriori corrected semi-empirical contribution 1/r6 Grimme dispersion correction (D2) was used [28]. The valence electrons were treated explicitly, whereas the core-electrons were treated using the Projector Augmented Wave (PAW) pseudopotentials [29], as implemented in the Quantum Espresso Plane Wave package version 6.4.1 [30] The complete electronic configuration of Mo is [Kr]4d^5^5s^1^, of Pb is [Xe]6s^2^4f^14^5d^10^6p^2^ and S is [Ne]3s^2^3p^4^. In the present work, the valence configurations of Mo, Pb, and S are taken to be 4s^2^5s^1^4p^6^5p^0^4d^5^, 6s^2^6p^2^5d^10^, and of 3s^2^3p^4^, respectively.

The *H* layer is a rectangular cell of MoS_2_ monolayer with 12 atoms per unit cell and the *Q* layer is the rectangular PbS bilayer with 8 atoms per unit cell. The 2D MoS_2_-PbS heterostructure consists of 1 × 1 cells each of *H* and *Q* layers. For the constituent atoms of the heterostructure, the valence electrons were taken to be 14, 14, and 6 for Pb, Mo, and S atoms, respectively.

In our calculations, the wave function and density cut-offs were set to be 40 and 400 *Ry*, respectively. Atomic positions in the periodic unit cell were allowed to relax until the total forces on each atom were less than ~10^−4^ *e*V/Å, and the self-consistency tolerance was set to 10^−6^ *Ry*. A vacuum spacing of about ~15 Å was employed along the crystallographic c-axis. For the density of states (DOS) calculations, the integration of the Brillouin zone was made on a Monkhorst-Pack grid of (2 × 2 × 1) k-points in the reciprocal space [31]. The room-temperature electron and hole mobilities were calculated by applying a phonon-limited scattering model including the anisotropic characteristics of effective mass following the expression, ES1 (electronic supplementary information (ESI)) given by Bardeen and Shockley [32]. It is worth mentioning that the expression, ES1 (ESI) gives only an estimated value of the carrier mobility in a given material [33].

## 3. Results and Discussion

### 3.1. Structural Properties

Considering the complexities of merelaniite’s incommensurate structure and the chemical formula Mo_4_Pb_4_VSbS_15_ [22], we focused on a highly simplified model of a 2D heterostructure that consists of one MoS_2_ (*H*) and two PbS (*Q*) layers (Figure S1a). It is worth noting that a similar assumption was made in the computational investigation of the properties of the 2D franckeite-inspired heterostructures composed of SnS2-based (i.e., H) and PbS-based (i.e., *Q*) layers [15]. In our model, the 2D MoS_2_-PbS heterostructure is simulated by a rectangular unit cell for which the lattice parameters, a and b, are calculated to be 5.64 and 6.40 Å, respectively at the PBE (DFT) + D2 level of theory. Note that the calculated lattice parameters, a, b, and c of the bulk MoS_2_-PbS are 5.67, 6.22, and 12.73 Å, showing a good agreement with the corresponding experimental values of merelaniite [24]. In the 2D MoS_2_-PbS heterostructure, the *H*-layer remains nearly strain-free while the *Q* layer becomes strained whereby its lattice constant contracts ≈8% along with *a* and expands ≈6% along *b* in the unit cell. Furthermore, the *Q* layer is not atomically flat and its non-planar configuration can be described by the buckling height (***δ***_z_) and buckling angle (***α***) along the z-direction (i.e., perpendicular to the plane) as shown in Figure 1. The structural parameters of the 2D MoS_2_-PbS heterostructure are compared with those of the corresponding bulk MoS_2_-PbS material in Table 1.

The calculated results predict the stability of the 2D MoS_2_-PbS heterostructure with a binding energy of −0.75 eV compared to a value of −1.57 eV for the bulk MoS_2_-PbS, which is defined as (E*_HQ_*−E*_H_*–E*_Q_*). Note that the heterostructure is not bound at the DFT level of the theory with binding energy of 0.03 eV. Thus, the inclusion of the D2 term appears to be essential to predict the stability of such 2D heterostructures. 

The interplanar distance between the *H* and *Q* layer (R_S(*H*)–Pb(*Q*)_) is 3.07 Å, which is about 7% smaller than that calculated in the bulk material (Table 1). For the *Q* layer, the buckling-height and buckling-angle values are slightly greater than the corresponding bulk values. This is expected due to the lack of periodicity along the z-axis for a 2D heterostructure. It is also worth mentioning that R_S(*H*)–Pb(*Q*)_ appears to be a representative interplanar distance of the constituent layers interacting via the vdW forces in the 2D MoS_2_-PbS heterostructure. We note here that previous calculations on graphene/SnO heterostructures using either PBE-D2 or optB88-vdW functional forms find that the stability of such heterostructures does not sensitively depend on the way we describe the vdW interactions [34]. 

To evaluate the mechanical stability, we calculate the elastic constants for the 2D MoS_2_-PbS heterostructure. The elastic constants for the rectangular symmetry must satisfy the following necessary and sufficient elastic stability conditions, also called Born stability conditions [35]:

C_11_ > 0 & C_33_ > 0 & C_11_C_22_ > C^2^_12_ (1)

or

(2) $$\lambda_{I}=\frac{1}{2}(C_{11}+C_{22}+\sqrt{4C_{12}^{2}-{\left(C_{11}-C_{22}\right)}^{2}}\;)\;\lambda_{II}=\frac{1}{2}(C_{11}+C_{22}-\sqrt{4C_{12}^{2}-{\left(C_{11}-C_{22}\right)}^{2}\;})\;\lambda_{III}=C_{33}>0$$

For the MoS_2_-PbS heterostructure, the calculated elastic constants are C_11_ = 134.3. C_22_ = 94.5, C_33_ = 36.1, and C_12_ = 26.9 GPa, thus satisfying the above conditions for mechanical stability.

### 3.2. Electronic Properties

**Figure 2:**
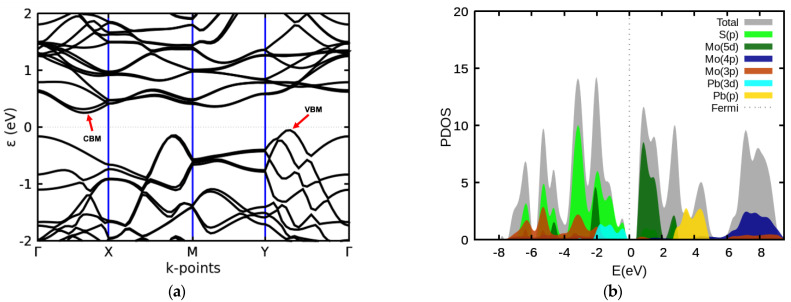
Calculated (**a**) band structure and (**b**) partial density of states of the 2D *QH* MoS_2_-PbS heterostructure. The conduction band minimum (CBM) and valence band maximum (VBM) are indicated in (**a**).

Figure 2a shows the electronic band structures of the 2D MoS_2_-PbS heterostructure, in which the top of the valence band is associated with the *Q* layer and the bottom of the conduction band is associated with the *H* layer. The calculated minimum energy gap is ≈0.2 eV and is predicted to be indirect, with the top of the valance band being at a point between Y and Γ and the bottom of the conduction band at a point between Γ and X. Interestingly, the bandgap of the free-standing *H*-layer (i.e., MoS_2_ monolayer) is calculated to be 1.8 eV and that of the free-standing *Q* layer (i.e., PbS bilayer) is calculated to be 1.4 eV (Figure S2a,b, (ESI)). This is what we expected because when two layers with different work functions come into contact with each other, the charge redistribution and band bending occur to equalize the work functions between the layers in a heterostructure. Moreover, the weak interlayer coupling also leads to changes in band positions of the constituent monolayers of the heterostructures [36] (Figure S1b).

The interaction of constituent layers via weak vdW forces is affirmed by the partial density of states, which shows that the PbS valence band, constituting the Pb-*d* and S-*p* orbitals, move upward towards the Fermi level while the MoS_2_ conduction band, comprised of Mo-*d* and S-*p* orbitals, moves downward towards the Fermi level for the 2D heterostructure (Figure S2c, (ESI) and Figure 2b). On the other hand, S-*p* and Mo-*d* orbitals appear to form a band at about 2 eV below the Fermi energy (Figure 2b). Figure S2c shows the upshifting of S-*p* and Pb-*d* orbitals (dominant valence band orbitals) and the downshifting of the Mo-*d* orbitals (dominant conduction band orbitals) in the heterostructure relative to the individual monolayers. 

We should stress here that our calculations did not take account of the spin-orbit coupling (SOC) effects for Pb atoms. However, it has previously been demonstrated that the inclusion of SOC effects does not significantly change the band structure of the 2D franckeite-inspired heterostructure [15].

Since the interlayer interaction between the *H* and *Q* layers is dominated by the vdW interactions, we expect a negligible charge transfer between the two constituents. This is affirmed by the calculated Lowdin’s charges, which suggest that ≈0.05 electron transfers from the *H* layer to the *Q* layer in the 2D heterostructure. Interestingly, such a small charge transfer still induces an electrostatic potential at the interface (Figure S3 (ESI)) that acts as a barrier between the *H* and *Q* layers, thereby promoting separation of electron-hole pairs in the heterostructure. The 2D MoS_2_-PbS heterostructure can therefore be considered for optoelectronic applications owing to its narrow bandgap of about 0.2 eV calculated at the PBE (DFT)-D2 level of theory. It is worth mentioning that calculations using a relatively accurate exchange and correlational functional form, the hybrid nonlocal exchange-correlation functional yield the bandgap to be about 0.45 eV (Figure S4, ESI) [28,37]. Such narrow bandgap materials are usually good for thermoelectric applications [38,39].

Carrier transport properties were investigated using the expression ES1 (ESI), which depends upon the in-plane stiffness, carrier effective mass, and deformation potential values listed in Table 2. These values were calculated by applying a strain ranging from −2% to +2% in steps of 0.5% to the 2D MoS_2_-PbS heterostructure. Note that the deformation potential of the electrons (holes) can be obtained by linear fitting of the curve of the conduction band edge (valance band edge) versus applied strain for electrons (holes). On the other hand, the in-plane stiffness can be obtained by the quadratic fitting of the expression, ES2 (ESI). We find that the in-plane stiffness for the 2D MoS_2_-PbS heterostructure exhibits anisotropic characteristics that are likely due to the *Q*. layer (Table 1). We estimate the effective mass of electrons or holes by the quadratic fitting of bands near the conduction band minimum (CBM) or valence band maximum (VBM), respectively. A smaller value of a carrier effective mass leads to higher carrier mobility. We find that the carrier effective masses to be anisotropic with holes being heavier along the x-direction and electrons being heavier along the y-direction. 

**Figure 3:**
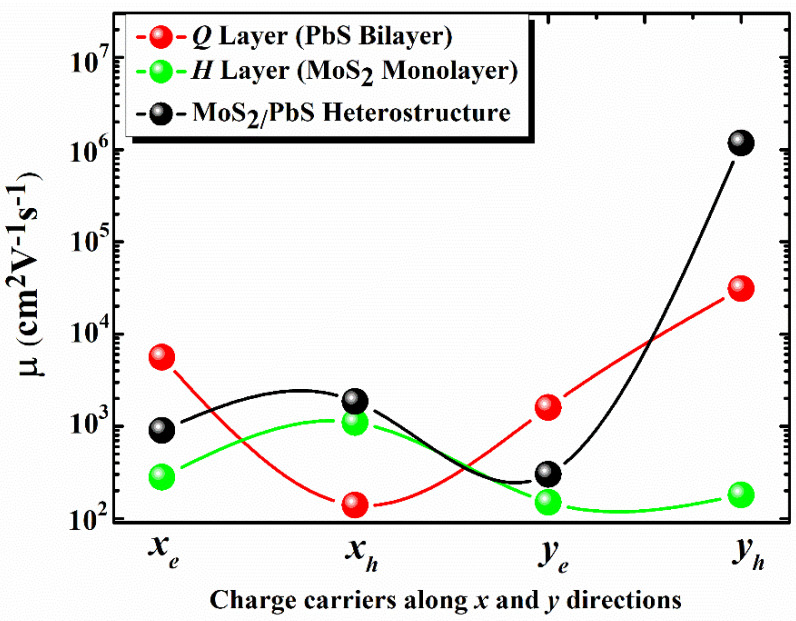
Calculated electron (e) and hole (h) mobilities of the 2D MoS_2_-PbS heterostructure and the free-standing PbS and MoS_2_ monolayers.

The calculated results show the 2D MoS_2_-PbS heterostructure to be p-type with the hole mobility being significantly higher than the electron mobility. Moreover, the mobility anisotropy, which is calculated as R_a_ = max(*μ_x_, μ_y_*)/min(*μ_x_, μ_y_*), is predicted to be 3.5 and 634.4 for electrons and holes, respectively in the heterostructure. It is interesting to note that the carrier mobility of the heterostructure is essentially governed by the constituent layers; the hole mobility is governed by the *Q* layer whereas the electron mobility is governed by the *H* layer, as shown in Figure 3. This is what we expected, since the constituent layers in the 2D MoS_2_-PbS heterostructure are bonded by the weak vdW forces, which do not significantly alter the dispersion of the valence or conduction bands of the constituents (Figure S5, ESI). It is to be noted that the calculated electron mobility of the MoS_2_ monolayer (Table 2) is comparable to previously reported experimental values, which are within the 0.5–220 cm^2^V^−1^s^−1^ range depending upon the different measuring techniques. Single-layer MoS_2_ fabricated using mechanical peeling or chemical exfoliation techniques and electrically contacted using electron-beam lithography on Si/SiO_2_ substrate (which also acts as the gate dielectric) can show field-effect mobilities of up to tens of cm^2^V^−1^s^−1^. The mobility can be improved by depositing a high-*κ* dielectric material such as HfO_2_, which overpowers the Coulomb scattering due to the high- *κ* environment and modifies the phonon dispersion giving high carrier mobility of ~200 cm^2^V^−1^s^−1^ [40,41,42,43].

## 4. Conclusions

In summary, the DFT calculations have shown that a merelaniite-inspired MoS_2_-PbS heterostructure can be formed from the corresponding bulk MoS_2_-PbS material. The heterostructure is predicted to be p-type with a bandgap of about 0.45 eV obtained at the HSE06-DFT level of theory. We found that the carrier mobility in the heterostructure is governed by the constituent layers as the heterostructure is weakly bonded by vdW forces. Moreover, the hole mobility is predicted to be relatively high (~10^6^ cm^2^V^−1^s^−1^), which can be useful for nanoscale devices. Considering the recent fabrication and characterization of 2D franckeite-inspired heterostructures, we expect our theoretical work to motivate experimentalists in fabricating the 2D merelaniite heterostructures for energy-related applications.

## Figures and Tables

**Table 1:** Calculated bond distances (in Å) and buckling parameters of the 2D MoS_2_-PbS heterostructure consisting of a MoS_2_ monolayer (*H*) and a PbS bilayer (*Q*). The corresponding values of the bulk MoS_2_-PbS material are also listed.

Computed Parameter	Bond Distances (Å)	
	2D MoS_2_-PbS Heterostructure	Bulk MoS_2_-PbS
R_S(*H*)–Pb(*Q*)_-interplanar	3.07	3.30
R_S(*H*)–Mo(*H*)_-intraplanar	2.41	2.41
R_S(*Q*)–Pb(*Q*)_-intraplanar	2.89	2.88
R_S(*Q*)–Pb(*Q*)_-interplanar	2.73	3.16
*Q*-layer buckling height (*δ*_z_)	0.71	0.51
*Q*-layer buckling angle (*α*)	13.5◦	9.8◦

**Table 2:** Calculated in-plain stiffness (C_2D_), effective mass (m*), deformation potential (*E*_1_), and mobility (*µ*) of 2D MoS_2_-PbS heterostructure and (free-standing) constituent layers;) and *xh* (*yh*), respectively, are electrons and holes along x-direction (y-direction).

		*C*_2D_ (N/m)	m* (m_e_)	*E*_1_ (eV)	*µ* (10^3^ cm^2^V^−1^s^−1^)
*H-Q* heterostructure	*xe*	276.3	0.36	5.83	0.90
	*xh*	276.3	0.91	3.48	1.86
	*ye*	190.1	0.83	5.50	0.30
	*yh*	190.1	0.09	0.36	1180.0
(free standing) *H* layer (MoS_2_ monolayer)	*xe*	167.5	0.42	7.8	0.28
	*xh*	167.5	0.42	2.5	1.10
	*ye*	164.1	0.83	6.9	0.15
	*yh*	164.1	3.9	2.0	0.18
(free standing) *Q* layer (PbS bilayer)	*xe*	112.0	0.16	3.00	5.6
	*xh*	112.0	2.4	4.00	0.14
	*ye*	26.6	0.56	1.45	1.6
	*yh*	26.6	0.09	0.66	31.0

## Data Availability

Data is contained within the article or Supplementary Material. The data presented in this study are available in the Supplementary section and can also be obtained from the corresponding author.

## Supplementary Materials

The following are available online at https://www.mdpi.com/article/10.3390/ma14071649/s1, Figure S1. (a) The unit cell of a 2D heterostructure of MoS_2_-PbS, and (b) the charge density difference plot for the MoS_2_-PbS heterostructure. (blue for depletion and red for accumulation). Isovalue 0.01 e/Å^3^., Figure S2. Calculated band structure of the (free standing) (a) *H* (i.e. MoS_2_ monolayer) and (b) *Q* (i.e. PbS bilayer) layers. (c) The partial density of states for the *H* layer (Red), *Q* layer (Green), and the 2D MoS_2_/PbS heterostructure (Blue); (c)-(i) p-orbitals of the S atoms, (c)-(ii) d-orbitals of the Mo atoms and (c)-(iii) d-orbitals of the Pb atoms., Figure S3. The calculated profile of the planar averaged self-consistent electrostatic potential of the 2D merelaniite heterostructure as a function of position., Figure S4. (a) Calculated PBE (DFT)+D2 band structure and (b) HSE06 band structure obtained at the PBE (DFT)+D2 structural configuration. Figure S5. Band energy (*E*_edge_) of CBM and VBM as a function of lattice dilation e along x and y directions for 2D MoS_2_-PbS heterostructure.

## References

[1] Novoselov K.S., Geim A.K., Morozov S.V., Jiang D., Zhang Y., Dubonos S.V., Grigorieva I.V., Firsov A.A. (2004). Electric Field Effect in Atomically Thin Carbon Films. Science.

[2] Bhimanapati G.R., Lin Z., Meunier V., Jung Y., Cha J., Das S., Xiao D., Son Y., Strano M.S., Cooper V.R. (2015). Recent Advances in Two-Dimensional Materials beyond Graphene. ACS Nano.

[3] Aïssa B., Memon N.K., Ali A., Khraisheh M.K. (2015). Recent Progress in the Growth and Applications of Graphene as a Smart Material: A Review. Front. Mater..

[4] Novoselov K.S., Fal V.I., Colombo L., Gellert P.R., Schwab M.G., Kim K. (2012). A Roadmap for Graphene. Nature.

[5] Chhowalla M., Shin H.S., Eda G., Li L.-J., Loh K.P., Zhang H. (2013). The Chemistry of Two-Dimensional Layered Transition Metal Dichalcogenide Nanosheets. Nat. Chem..

[6] Chhowalla M., Liu Z., Zhang H. (2015). Two-Dimensional Transition Metal Dichalcogenide (TMD) Nanosheets. Chem. Soc. Rev..

[7] Wang Q.H., Kalantar-Zadeh K., Kis A., Coleman J.N., Strano M.S. (2012). Electronics and Optoelectronics of Two-Dimensional Transition Metal Dichalcogenides. Nat. Nanotechnol..

[8] Geim A.K., Grigorieva I.V. (2013). Van der Waals Heterostructures. Nature.

[9] Novoselov K.S., Mishchenko A., Carvalho A., Neto A.H.C. (2016). 2D Materials and van der Waals Heterostructures. Science.

[10] Jariwala D., Marks T.J., Hersam M.C. (2017). Mixed-Dimensional van der Waals Heterostructures. Nat. Mater..

[11] Lafond A., Nader A., Moëlo Y., Meerschaut A., Briggs A., Perrin S., Monceau P., Rouxel J. (1997). X-ray Structure Determination and Superconductivity of a New Layered Misfit Compound with a Franckeite-like Stacking, [(Pb,Sb)S]_2.28_ NbS_2_. J. Alloy. Compd..

[12] Kaden R., Wagner G., Sturm C., Schmidt-Grund R., von Wenckstern H., Prager A., Bente K., Grundmann M. (2010). Synthesis and Physical Properties of Cylindrite Micro Tubes and Lamellae. Phys. Status Solidi..

[13] Makovicky E., Petricek V., Dusek M., Topa D. (2008). Crystal Structure of a Synthetic Tin-Selenium Representative of the Cylindrite Structure Type. Am. Mineral..

[14] Sachdev S.C., Chang L.L.Y. (1975). Phase Relations in the System Tin-Antimony-Lead Sulfides and the Synthesis of Cylindrite and Franckeite. Econ. Geol..

[15] Molina-Mendoza A.J., Giovanelli E., Paz W.S., Niño M.A., Island J.O., Evangeli C., Aballe L., Foerster M., Van Der Zant H.S.J., Rubio-Bollinger G. (2017). Franckeite as a Naturally Occurring van der Waals Heterostructure. Nat. Commun..

[16] Velicky M., Toth P.S., Rakowski A.M., Rooney A.P., Kozikov A., Woods C.R., Mishchenko A., Fumagalli L., Yin J., Zólyomi V. (2017). Exfoliation of Natural van der Waals Heterostructures to a Single Unit Cell Thickness. Nat. Commun..

[17] Ray K., Yore A.E., Mou T., Jha S., Smithe K.K.H., Wang B., Pop E., Newaz A.K.M. (2017). Photoresponse of Natural van der Waals Heterostructures. ACS Nano.

[18] Wang S., Kuo K.H. (1991). Crystal Lattices and Crystal Chemistry of Cylindrite and Franckeite. Acta Crystallogr. Sect. A Found. Crystallogr..

[19] Makovicky E., Hyde B.G. (1992). Incommensurate, Two-Layer Structures with Complex Crystal Chemistry: Minerals and Related Synthetics. Materials Science Forum.

[20] Matzat E. (1979). Cannizzarite. Acta Crystallogr. Sect. B Struct. Crystallogr. Cryst. Chem..

[21] Moëlo Y., Makovicky E., Mozgova N.N., Jambor J.L., Cook N., Pring A., Paar W., Nickel E.H., Graeser S., Karup-Møller S. (2008). Sulfosalt Systematics: A Review. Report of the Sulfosalt Sub-Committee of the IMA Commission on Ore Mineralogy. Eur. J. Mineral..

[22] Lian X., Niu M., Huang Y., Cheng D. (2018). MoS_2_-CdS Heterojunction with Enhanced Photocatalytic Activity: A First Principles Study. J. Phys. Chem. Solids.

[23] Li X.-B., Guo P., Zhang Y.-N., Peng R.-F., Zhang H., Lui L.-M. (2015). High Carrier Mobility of Few-Layer PbX (X = S, Se, Te). J. Mater. Chem. C.

[24] Jaszczak J., Rumsey M., Bindi L., Hackney S., Wise M., Stanley C., Spratt J. (2016). Merelaniite, Mo_4_Pb_4_VSbS_15_, a New Molybdenum-Essential Member of the Cylindrite Group, from the Merelani Tanzanite Deposit, Lelatema Mountains, Manyara Region, Tanzania. Minerals.

[25] Yang Y., He H., Tan W., Tao Q., Yao J., Xian H., Li S., Xi J., Zhu J., Xu H. (2021). Incorporation of incompatible trace elements into molybdenite: Layered PbS precipitates within molybdenite. Am. Mineral. Pap..

[26] Li J., Yang K., Du L., Yi J., Huang J., Zhang J., He Y., Huang B., Miao L., Zhao C. (2020). Nonlinear Optical Response in Natural van der Waals Heterostructures. Adv. Opt. Mater..

[27] Hammer B., Hansen L.B., Nørskov J.K. (1999). Improved Adsorption Energetics within Density-Functional Theory Using Revised Perdew-Burke-Ernzerhof Functionals. Phys. Rev. B.

[28] Grimme S., Antony J., Ehrlich S., Krieg H. (2010). A Consistent and Accurate Ab Initio Parametrization of Density Functional Dispersion Correction (DFT-D) for the 94 Elements H-Pu. J. Chem. Phys..

[29] Kresse G., Joubert D. (1999). From Ultrasoft Pseudopotentials to the Projector Augmented-Wave Method. Phys. Rev. B.

[30] Giannozzi P., Andreussi O., Brumme T., Bunau O., Nardelli M.B., Calandra M., Car R., Cavazzoni C., Ceresoli D., Cococcioni M. (2017). Advanced Capabilities for Materials Modelling with Quantum ESPRESSO. J. Phys. Condens. Matter.

[31] Evarestov R.A., Smirnov V.P. (2004). Modification of the Monkhorst-Pack Special Points Meshes in the Brillouin Zone for Density Functional Theory and Hartree-Fock Calculations. Phys. Rev. B.

[32] Bardeen J., Shockley W. (1950). Deformation Potentials and Mobilities in Non-Polar Crystals. Phys. Rev..

[33] Gaddemane G., Vandenberghe W.G., van de Put M.L., Chen S., Tiwari S., Chen E., Fischetti M.V. (2018). Theoretical Studies of Electronic Transport in Mono- and Bi-Layer Phosphorene: A Critical Overview. arXiv.

[34] Guo Q., Wang G., Pandey R., Karna S.P. (2018). Robust Band Gaps in the Graphene/Oxide Heterostructure: SnO/Graphene/SnO. Phys. Chem. Chem. Phys..

[35] Maździarz M. (2019). Comment on ‘The Computational 2D Materials Database: High-throughput modeling and discovery of atomically thin crystals’. 2D Mater..

[36] Kaur S., Kumar A., Srivastava S., Tankeshwar K. (2017). van der Waals heterostructures based on allotropes of phosphorene and MoSe 2. Phys. Chem. Chem. Phys..

[37] Heyd J., Scuseria G.E., Ernzerhof M. (2003). Hybrid Functionals Based on a Screened Coulomb Potential. J. Chem. Phys..

[38] Bahk J.-H., Shakouri A. (2016). Minority Carrier Blocking to Enhance the Thermoelectric Figure of Merit in Narrow-Band-Gap Semiconductors. Phys. Rev. B.

[39] Kim I.-H. (2018). Narrow Bandgap Thermoelectric Semiconductors. J. Korean Phys. Soc..

[40] Krymowski K. (2015). Electronic Mobilities of Two-Dimensional Transition Metal Dichalcogenides.

[41] Radisavljevic B., Radenovic A., Brivio J., Giacometti V., Kis A. (2011). Single-Layer MoS_2_ Transistors. Nat. Nanotechnol..

[42] Ayari A., Cobas E., Ogundadegbe O., Fuhrer M.S. (2007). Realization and Electrical Characterization of Ultrathin Crystals of Layered Transition-Metal Dichalcogenides. J. Appl. Phys..

[43] Fivaz R., Mooser E. (1967). Mobility of Charge Carriers in Semiconducting Layer Structures. Phys. Rev..

