# Supplementary material for: First-Principles Study of a MoS2-PbS van der Waals Heterostructure Inspired by Naturally Occurring Merelaniite
Source: Materials (Basel). 2021 Mar 27;14(7):1649. doi: 10.3390/ma14071649 (PMC8037089; doi:10.3390/ma14071649)
Supplement: Supplementary file 1 [file materials-14-01649-s001.pdf]

materials

Supplementary Materials

First-Principles Study of a MoS_2-PbS van der Waals Heterostructure Inspired by Naturally Occurring Merelaniite

Gemechis D. Degaga [1], Sumandeep Kaur [1], Ravindra Pandey [1,*] and John A. Jaszczak [1]

[1] Department of Physics, Michigan Technological University, Houghton, MI 49931, USA; gdegaga@mtu.edu (G.D.D); sumandek@mtu.edu (S.K.); jaszczak@mtu.edu (J.A.J.)
* Correspondence: pandey@mtu.edu

Citation: Degaga, G.D.; Kaur, S.; Pandey, R.; Jaszczak, J.A. First-Principles Study of a MoS_2-PbS van der Waals Heterostructure Inspired by Naturally Occurring Merelaniite. *Materials* **2021**, *14*, 1649. https://doi.org/10.3390/ma14071649

Academic Editor: Shirley Chiang

Received: 14 February 2021
Accepted: 24 March 2021
Published: 27 March 2021

Publisher's Note: MDPI stays neutral with regard to jurisdictional claims in published maps and institutional affiliations.

Copyright: © 2021 by the authors. Submitted for possible open access publication under the terms and conditions of the Creative Commons Attribution (CC BY) license (http://creativecommons.org/licenses/by/4.0/).

1.0. Electronic Properties: Band Structure and Density of States

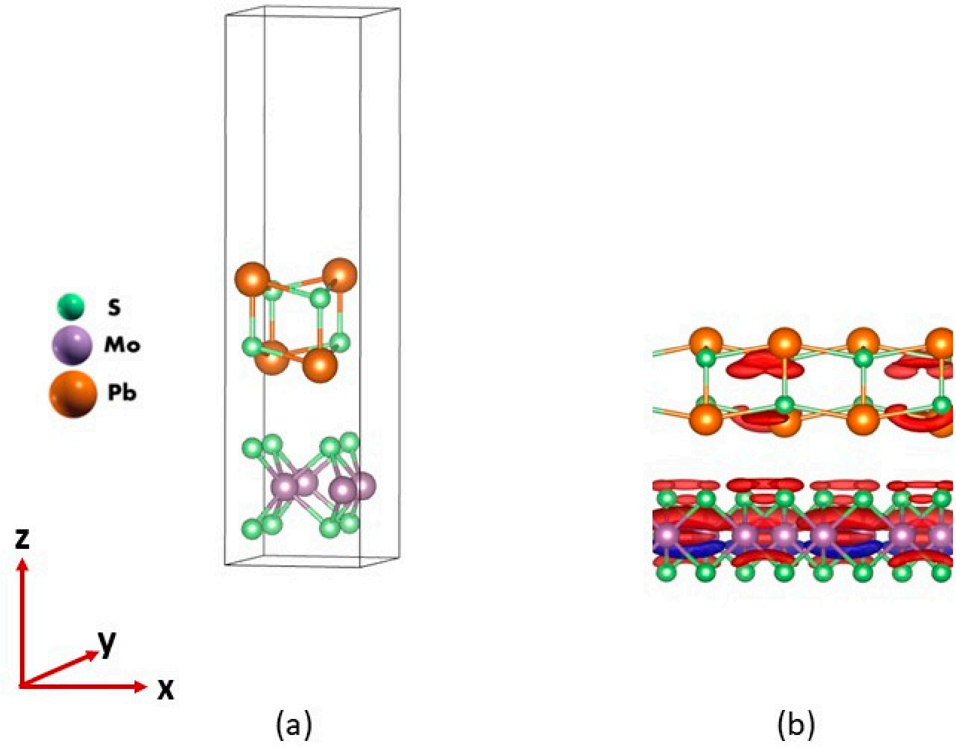

Figure S1. (**a**)The unit cell of a 2D heterostructure of MoS_2-PbS, and (**b**) the charge density difference plot for the MoS_2-PbS heterostructure. (blue for depletion and red for accumulation). Isovalue 0.01 e/Å^3.

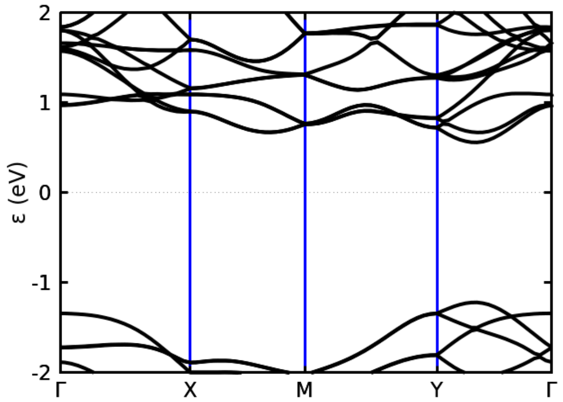

(**a**)

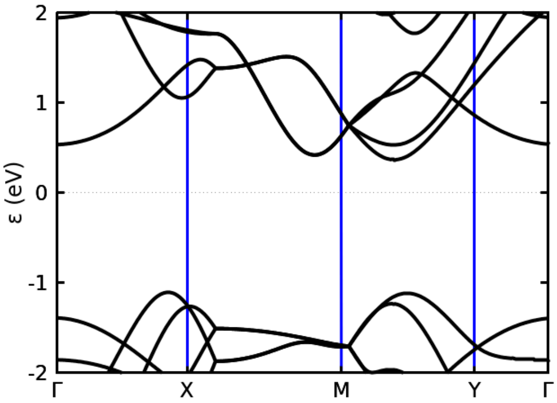

(**b**)

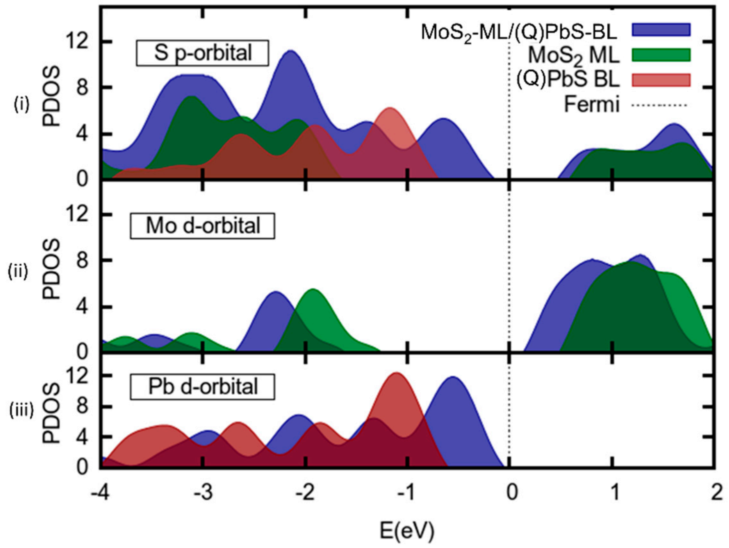

(**c**)

Figure S2. Calculated band structure of the (free standing) (**a**) *H* (i.e. MoS_2 monolayer) and (**b**)*Q* (i.e. PbS bilayer) layers. (**c**) The partial density of states for the *H* layer (Red), *Q* layer (Green), and the 2D MoS_2-PbS heterostructure (Blue); (**c**)-(**i**) p-orbitals of the S atoms, (**c**)-(**ii**) d-orbitals of the Mo atoms and (**c**)-(**iii**) d-orbitals of the Pb atoms.

2.0. The Electrostatic Potential:

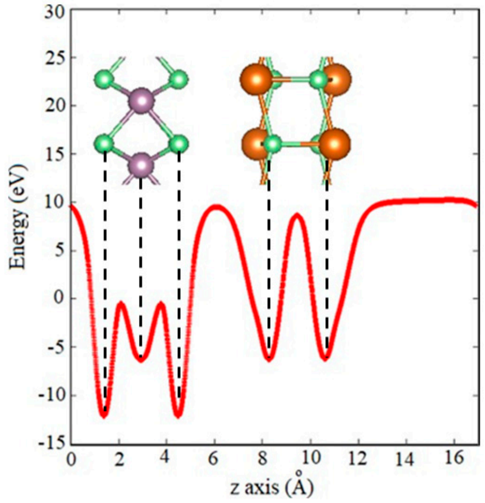

Figure S3. The calculated profile of the planar averaged self-consistent electrostatic potential of the 2D merelaniite heterostructure as a function of position.

3.0. VASP Calculations

In general, the HSE06 calculations are about two orders of magnitude computationally expensive in Quantum espresso than those in VASP [1]. Therefore, we have used the VASP code to perform HSE06 calculations on the 2D MoS_2-PbS heterostructure [2,3] It is worth mentioning that the PBE (DFT) band structure calculated using VASP is nearly the same as obtained by the Quantum espresso. In VASP calculations, the energy cutoff was kept at 400 eV and the k-point grid was 7×7×1. The energy and forces were converged to 10^{-5} eV and 10^{-2} eV/Å, respectively.

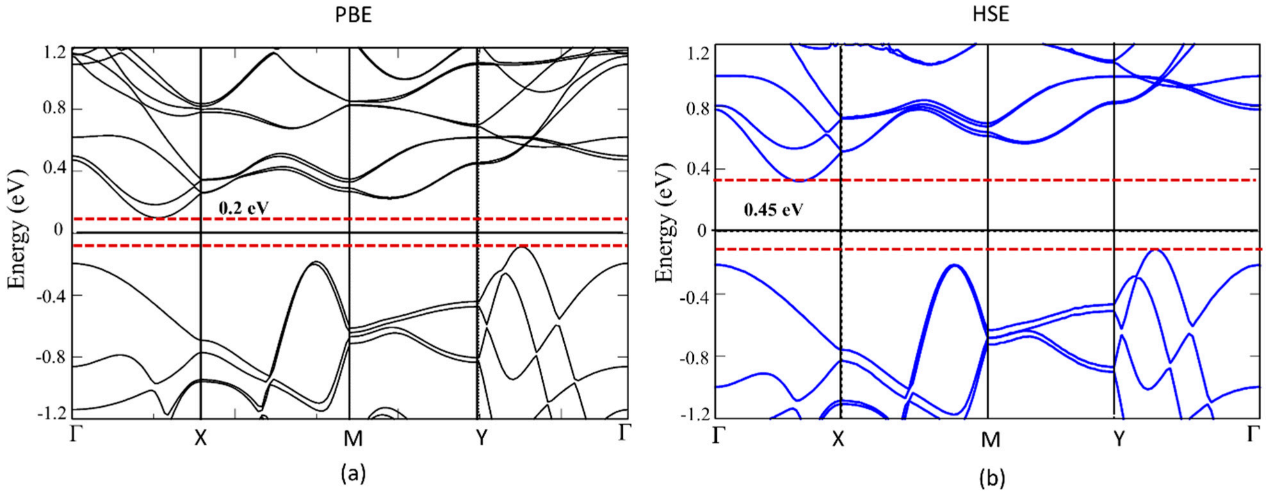

Figure S4. (**a**) Calculated PBE (DFT)+D2 band structure and (**b**) HSE06 band structure obtained at the PBE (DFT)+D2 structural configuration.

4.0. Calculations of Carrier Mobility

The room-temperature carrier mobilities (μ_{2D}) were calculated by applying a phonon-limited scattering model including the anisotropic characteristics of effective mass following the expression, Equation S1 given by Bardeen and Shockley [4].

$$\mu_{2D} = \frac{e\hbar^3 C_{2D}}{K_B T m^* m_a^* E_i^2} \quad \text{(S1)}$$

where e is the electronic charge, $\hbar$ is the reduced Plank's constant, T is the temperature, kB is the Boltzmann constant, m* is the effective mass in either along x- or y- direction, ma* is the average effective mass given by $\sqrt{m_x^* m_y^*}$. E_i is the deformation-potential constant calculated using the expression, $E_i = \frac{dE_{\text{edge}}}{de}$ where E_{edge} is the energy of the CBM (VBM) for electrons (holes) and e = $\Delta l/l_0$, and $\Delta l/l_0$ is the strain/lattice dilation along x- or y-direction.

The in-plane elastic modulus, C_{2D} was calculated using:

$$(E - E_0)/S_0 = C_{2D}\,(\Delta\, l/l_0)^2/2 \quad \text{(S2)}$$

where $E - E_0$ represents total energy change, S_0 is the area of the 2D cell and, $\Delta l/l_0$ is the the strain along x or y-direction [5]. It is worth mentioning that the expression, Equation S1 (ESI) gives only an estimated value of the carrier mobility in a given material [6].

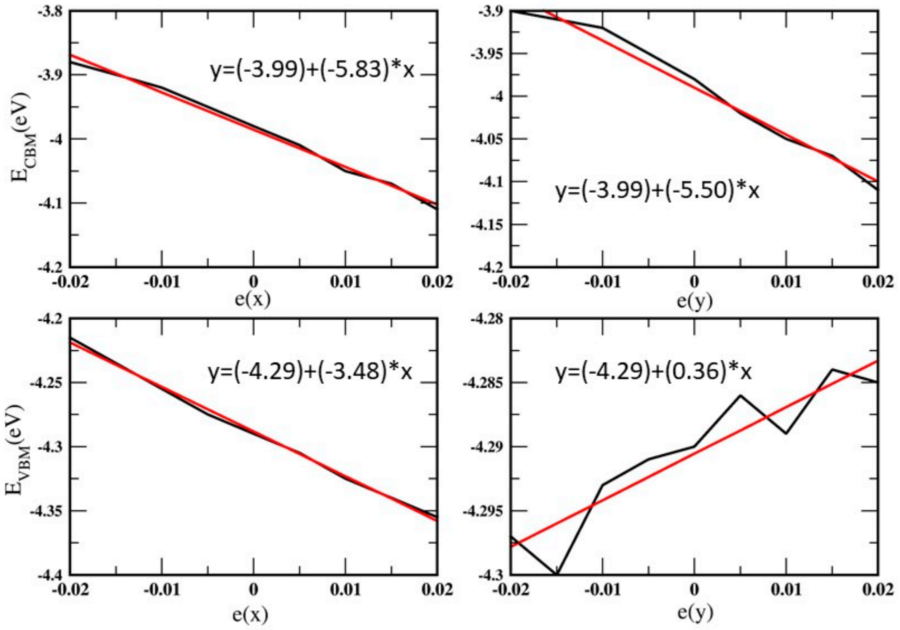

Figure S5. Band energy (E_{edge}) of CBM and VBM as a function of lattice dilation e along x and y directions for 2D MoS_2-PbS heterostructure.

5.0. Structural Parameters for MoS_2-PbS Heterostructure

Lattice Vectors (Å)		
5.4739656448	0.0000000000	0.0000000000
0.0000000000	6.3207879066	0.0000000000
0.0000000000	0.0000000000	24.0000000000

Atomic Positions (Cartesian Coordinates)		
0.912116	0.790098	1.365608
3.652712	2.364822	1.362597
0.91875	3.950492	1.35763
3.652712	5.536163	1.362597
0.916813	0.790098	4.532084
3.652164	2.377615	4.519615
0.913364	3.950492	4.492824
3.652164	5.52337	4.519615
0.908153	0.790098	8.504323
3.643472	0.790098	10.43171
0.909773	3.950492	10.49807
3.646854	3.950492	8.52363
4.56393	0.790098	2.948438
1.826947	2.36821	2.938384
4.562025	3.950492	2.930117
1.826947	5.532775	2.938384
0.907365	0.790098	11.12649
3.645749	0.790098	7.809825
0.908142	3.950492	7.883156
3.645267	3.950492	11.1454

References:

1. https://mattermodeling.stackexchange.com/questions/561/quantum-espresso-vs-vasp. (Accessed on January 15, 2021).
2. Heyd, J.; Scuseria, G. E.; Ernzerhof, M. Hybrid Functionals Based on a Screened Coulomb Potential. *J. Chem. Phys.* 2003, 118 (18), 8207–8215.
3. Kresse, Georg, and D. J. From Ultrasoft Pseudopotentials to the Projector Augmented-Wave Method. *Phys. Rev. B* 1999, 59 (3), 1758.
4. Bardeen, J.; Shockley, W. Deformation Potentials and Mobilities in Non-Polar Crystals. *Phys. Rev.* 1950, 80 (1), 72–80. https://doi.org/10.1103/PhysRev.80.72.
5. Gaddemane, G.; Vandenberghe, W. G.; van de Put, M. L.; Chen, S.; Tiwari, S.; Chen, E.; Fischetti, M. V. Theoretical Studies of Electronic Transport in Mono- and Bi-Layer Phosphorene: A Critical Overview. arXiv 2018, 98, 115416.
6. Qiao, Jingsi, Xianghua Kong, Zhi-Xin Hu, Feng Yang, and Ji, Wei. High-Mobility Transport Anisotropy and Linear Dichroism in Few-Layer Black Phosphorus. *Nat. Commun.* 2014, 5 (1), 1–7.
